# From mechanisms to markers: novel noninvasive EEG proxy markers of the neural excitation and inhibition system in humans

**DOI:** 10.1038/s41398-022-02218-z

**Published:** 2022-11-08

**Authors:** Jumana Ahmad, Claire Ellis, Robert Leech, Bradley Voytek, Pilar Garces, Emily Jones, Jan Buitelaar, Eva Loth, Francisco Páscoa dos Santos, Adrián F. Amil, Paul F. M. J. Verschure, Declan Murphy, Grainne McAlonan

**Affiliations:** 1 School of Human Sciences, University of Greenwich, London, UK; 2 Department of Forensic and Neurodevelopmental Sciences, Institute of Psychiatry, Psychology and Neuroscience, King’s College London, London, UK; 3 Sackler Institute for Translational Neurodevelopment, Institute of Psychiatry, Psychology & Neuroscience, King’s College London, London, UK; 4 Department of Neuroimaging, King’s College London, Institute of Psychiatry, Psychology and Neuroscience, King’s College London, London, UK; 5 Neurosciences Graduate Program, UC San Diego, La Jolla, CA USA; 6 Department of Cognitive Science, UC San Diego, La Jolla, CA USA; 7 Halıcıoğlu Data Science Institute, UC San Diego, La Jolla, CA USA; 8 Kavli Institute for Brain and Mind, UC San Diego, La Jolla, CA USA; 9 Roche Pharma Research and Early Development, Neuroscience and Rare Diseases, Roche Innovation Center Basel, Basel, Switzerland; 10 Centre for Brain and Cognitive Development, Birkbeck, University of London, London, UK; 11 Department of Cognitive Neuroscience, Donders Institute for Brain, Cognition, and Behaviour, Radboud University Medical Center, Nijmegen, The Netherlands; 12 Eodyne Systems SL, Barcelona, Spain; 13 Laboratory of Synthetic, Perceptive, Emotive and Cognitive Systems (SPECS), Institute for Bioengineering of Catalonia (IBEC), Barcelona, Spain; 14 Department of Information and Communications Technologies (DTIC), Universitat Pompeu Fabra (UPF), Barcelona, Spain; 15 Donders Institute for Brain, Cognition and Behavior, Radboud University, Nijmegen, The Netherlands; 16 South London and Maudsley, NHS Foundation Trust, London, UK

**Keywords:** Molecular neuroscience, Biomarkers

## Abstract

Brain function is a product of the balance between excitatory and inhibitory (E/I) brain activity. Variation in the regulation of this activity is thought to give rise to normal variation in human traits, and disruptions are thought to potentially underlie a spectrum of neuropsychiatric conditions (e.g., Autism, Schizophrenia, Downs’ Syndrome, intellectual disability). Hypotheses related to E/I dysfunction have the potential to provide cross-diagnostic explanations and to combine genetic and neurological evidence that exists within and between psychiatric conditions. However, the hypothesis has been difficult to test because: (1) it lacks specificity—an E/I dysfunction could pertain to any level in the neural system- neurotransmitters, single neurons/receptors, local networks of neurons, or global brain balance - most researchers do not define the level at which they are examining E/I function; (2) We lack validated methods for assessing E/I function at any of these neural levels in humans. As a result, it has not been possible to reliably or robustly test the E/I hypothesis of psychiatric disorders in a large cohort or longitudinal patient studies. Currently available, in vivo markers of E/I in humans either carry significant risks (e.g., deep brain electrode recordings or using Positron Emission Tomography (PET) with radioactive tracers) and/or are highly restrictive (e.g., limited spatial extent for Transcranial Magnetic Stimulation (TMS) and Magnetic Resonance Spectroscopy (MRS). More recently, a range of novel Electroencephalography (EEG) features has been described, which could serve as proxy markers for E/I at a given level of inference. Thus, in this perspective review, we survey the theories and experimental evidence underlying 6 novel EEG markers and their biological underpinnings at a specific neural level. These cheap-to-record and scalable proxy markers may offer clinical utility for identifying subgroups within and between diagnostic categories, thus directing more tailored sub-grouping and, therefore, treatment strategies. However, we argue that studies in clinical populations are premature. To maximize the potential of prospective EEG markers, we first need to understand the link between underlying E/I mechanisms and measurement techniques.

## Introduction

The coordination of excitatory (E) and inhibitory (I) activity is a fundamental property of brain function [1]—a specific ratio of E and I is thought to govern local and global network dynamics in the typically developing brain [2]. Throughout this article, we use the term ‘balance’—to refer to this optimal quantity of E and I that creates a state of low firing rates where excitatory activity does not either run away or die out after the arrival of an external stimulus/signal, as opposed to their equity. Converging evidence from genetic, postmortem, and preclinical studies suggests that disruptions to different components of excitatory and inhibitory processes are involved in a variety of clinical conditions, such as autism, schizophrenia, epilepsy, and generalized learning disabilities [3–10].

The theory that E/I imbalance underlies neurodevelopmental disorders has been difficult to test for two reasons. First, the E/I system is complex, yet most authors are unspecific in defining what they mean by E/I imbalance. For instance, E/I can be defined at multiple interacting levels, of the primary excitatory versus inhibitory neurotransmitters: glutamate and GABA, the E/I potentials within a neuron, or the E/I conductance between locally connected neurons versus entire networks of neurons at the system level. Hence, the failure to more precisely define E/ I balance clouds the synthesis of findings across different studies. Second, direct and indirect measurement of E/I at any given level in the living human brain is challenging. Methods such as Positron Emission Tomography (PET), Magnetic Resonance Spectroscopy (MRS), and Transcranial Magnetic Stimulation (TMS) provide useful indicators of E/I in terms of (respectively) receptor occupancy, neurotransmitter concentration, and neurotransmitter metabolism. However, they have a poor temporal resolution, are expensive and/or invasive; and (in the case of MRS and TMS) are generally limited to examining a relatively small number of brain regions at one time. As such, these methods alone offer an incomplete explanation of E/I.

In order to comprehensively assess brain E/I function in humans, we need proxy markers that can also tap into global E/I dynamics and methods that are scalable across research environments (i.e., translate from cellular, animal models to human research) and clinical settings. Much of the E/I research to date has come from animal models (please see refs. [11, 12]). Electrophysiological recordings are predominantly made up of aggregate postsynaptic and transmembrane currents [13], where excitatory and inhibitory currents dominate [14]. Traditionally, electrical potentials are termed the local field potential (LFP) when recorded using single electrodes from extracellular space; while electrocorticogram (ECoG) refers to intracranial recordings from the cortical surface; and electroencephalography (EEG) refers to recordings at the scalp [13]. When choosing a proxy marker of E/I, EEG has a number of advantages. EEG has an excellent temporal resolution to tap into real-time dynamics that are likely affected by subtle E/I fluctuations [13, 15]. Although often criticized for lacking spatial resolution, increasingly accurate source localization (i.e., using inverse models to estimate the likely brain dipoles that generated the signal recorded at the scalp) techniques do exist for high-density systems [16]. Furthermore, scalp EEG is a noninvasive, cheap methodology, and recordings can now take place in research settings, in the clinic, or at home. Combining EEG with techniques such as MRS and PET will allow us to gain a more complete picture of E/I functioning by leveraging the strengths of each method. However, for prospective EEG markers to be clinically translatable, we need to develop a comprehensive understanding of the neurobiological mechanisms that generate the signals measured. A more thorough understanding may help us bridge the gap between preclinical work (predominantly LFP, ECoG) and human EEG and will allow us to find EEG-compatible markers to facilitate clinical use.

To facilitate this, here we outline the concepts of E/I balance across cellular, local and global network scales. We then go on to propose a set of EEG features that may serve as ‘proxy markers’ for E/I at different neurobiological levels and consider the current evidence base for each one. Finally, we emphasize the need for biomarker validation in human EEG and consider whether, when validated, their application could help better stratify neurodevelopmental conditions and/or provide treatment targets to alleviate the burden of some neuropsychiatric conditions. Definitions and resources regarding how to extract and analyze these biomarkers have been provided in Table 1.

**Table 1:** Definition and resources for guidance on how to analyze each EEG marker.

EEG feature	Definition	Resources
Gamma power and Gamma frequency	Gamma refers to neural oscillations with a frequency between ~30 and 100+hz. Gamma power refers to the magnitude of the oscillation within this range. Gamma frequency refers to the number of cycles per second i.e., akin to the speed of the gamma oscillation.	Both fieldtrip and EEGlab are common Matlab toolboxes for analyzing gamma band activity. Please see https://eeglab.org/tutorials/08_Plot_data/Time-Frequency_decomposition.html for a tutorial and https://www.fieldtriptoolbox.org/workshop/madrid2019/tutorial_freq/ for tutorials
Beta power	Neural oscillations with a frequency between ~12 and 30 hz. Beta power refers to the magnitude of beta oscillations in the signal.	For readers interested in conducting beta power analysis, please follow the same tutorials provided for analyzing gamma band activity
Neural avalanches and Kappa coefficient	Neuronal avalanches refer to the organization of cascades of synchronous neural activity. The relationship between the size of neuronal events and the probability of finding an event follows a 1/f-like distribution. The Kappa coefficient describes the extent to which a neuronal avalanche follows a 1/f distribution.	For guidance on how to identify and calculate neuronal avalanches, please see [103, 161, 162], and for guidance on how to calculate k, please see [89, 101]
DFA Exponent and Functional E/I Balance	DFA exponents quantify the existence of long-range temporal correlations in the fluctuations in the amplitude of oscillatory signals. Critical systems show DFA exponents between 0.5 and 1. Functional E/I balance is an adaptation of DFA to give information on whether oscillatory signals come from networks that are either excitation or inhibition dominated	For guidance on how to perform DFA on oscillatory signals, please consult [100, 115]
Entropy	Entropy quantifies randomness in patterns of EEG and, in turn, how much information is in that signal. Brain entropy refers to the number of neural states in a signal.	For guidance on how to compute neuronal entropy, see: https://sapienlabs.org/measuring-entropy-in-the-eeg/; https://sapienlabs.org/the-impact-of-parameters-choices-on-eeg-entropy-measures/; Also see [163]
Microstates	EEG microstates refer to topographies in EEG which are shown to be stable for brief periods of time. Evidence from resting-state EEG has largely considered the same four microstate topographies, that explain most of the variance, and have been labeled A, B, C, and D [164].	For guidance on how to perform EEG microstates analysis, see [133]; Also see https://www.thomaskoenig.ch/index.php/work/ragu
Aperiodic 1/f signal	The aperiodic 1/f signal of EEG is a measure of non-oscillatory power across frequencies. This relationship between power and frequency, in many cases, is thought to follow a 1/ fχ distribution, and here aperiodic 1/f signal is quantified as the χ exponent.	The FOOOF model is an increasingly common method for extracting the aperiodic 1/f signal, see https://fooof-tools.github.io/fooof/index.html and [143]

## Excitation and inhibition in the brain

E and I are multiscale—each can be mediated and perturbed at multiple levels, from intracellular and single-cell to the local circuit and global network levels, and across various time scales [17–19]. At the cellular level, whether a neuron is excited or inhibited is determined by its state of polarization, which can be influenced by a variety of neurotransmitters. For the purposes of this review, we limit our discussion to the main excitatory (glutamate) and inhibitory (g-aminobutyric acid (GABA) neurotransmitters—since they have a leading role in regulating brain activity—although we acknowledge that in actuality, a complex combination of neurotransmitters is at play. Excitatory neurons are typically pyramidal cells, which project to other excitatory neurons and can form long-range synaptic connections across distant brain regions [20–22]. In neurons, excitatory neurotransmitters depolarize the postsynaptic neuron, making it more likely to fire. Inhibitory neurons e.g., parvalbumin (PV) and somatostatin (SS) neurons, form local, dense connections [23] and release GABA, which hyperpolarizes the postsynaptic cell, making it less likely to fire. At early stages of development, research in rodents suggests that activation of GABAA receptors generates postsynaptic membrane depolarization (i.e., an excitatory effect instead of hyperpolarization); but shortly after birth a ‘GABAA switch’ occurs—and GABAA leads to postsynaptic membrane hyperpolarization (e.g., [24]). However, exactly how and when this occurs in humans is unknown, but researchers have hypothesized that disruption/ delay in this process may underlie neurodevelopmental conditions [25]. Inhibition is also playing a role in the regulation of plasticity through shunting inhibition [26]. This involves removing depolarized currents generated by dendritic cells [27]. Shunting inhibition is thought to modulate spike time-dependent plasticity (STDP), for e.g., by adjusting synaptic strength based on the timing of neuronal firing [28], therefore dictating learning and information processing [29].

At the micro-level (single-cell), E/I can reflect the relative amount of excitatory glutamate versus inhibitory GABA synaptic inputs onto a single neuron [30, 31], or the quantity of excitatory and inhibitory synapses on individual cortical neurons [32] and their dynamic configuration along the dendrite through the characteristic length constant of the postsynaptic dendrite [33]. Accordingly, researchers using animal models may characterize E/I at this level by recording from the membrane using clamp recordings. Across a local population of cells, excitation and inhibition operate in unison where the excitatory current may increase while the inhibitory current decreases or the inhibitory current may decrease after a short time delay ([12, 34–38]). Specifically, it has been proposed that the direct coupling between E and I lead to a winnertake-all theta-gamma code [39]. The regulation of E/I balance can be seen to achieve homeostatic optimization supporting specific neuronal functions [11]. The way E/I balance is conceptualized at the meso-level (local populations of neurons) may be a composite of the relative concentration of excitatory versus inhibitory neurotransmitters, and the amount of excitatory versus inhibitory neurons or excitatory versus inhibitory receptors within any local brain region. Inferences at this level can be made by examining cell types within a region of postmortem tissue, or by assessing the relative concentration of neurotransmitters using microdialysis. In living humans, researchers may use proton MRS to obtain a somewhat coarse quantification of excitatory to inhibitory neurotransmitter concentration within a relatively large volume of the brain (typically cm3 and including both gray and white matter) [40].

At the macro-level E/I can be defined as the population-level activity, the result of large-scale interaction between neurons. This interaction can be examined within local regions or between more distal regions at the global network level. This latter scale of E/I reflects the recurrent connectivity from both excitatory and inhibitory neurons, which settle into a state of population activity that reflects the balance between the two [41, 42]. The activity of circuits at meso and macro-levels is a composite of this complex underlying neuronal activity—and so meso- and macro-scale processes also reflect neuronal excitation and inhibition at the microscale; for e.g., how EPSP and IPSP generate and reflect the properties that we see in the local field potential. This is likely true when we sum across multiple independent LFPs but also when we tap into specific networks i.e., coordinated long-range connectivity analysis. Having outlined the levels of inference, we suggest that future E/I research should explicitly specify the neuronal level that is being examined.

**Fig. 1:**
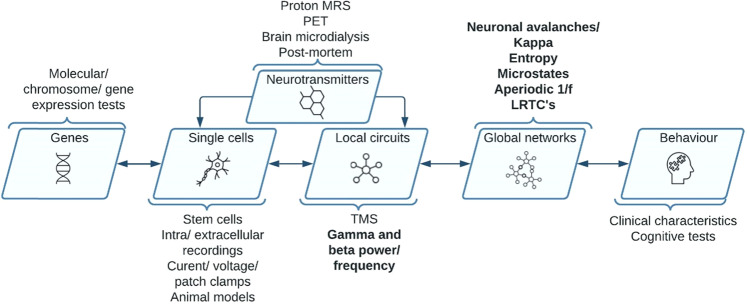
This figure describes the hierarchical levels of neural inference in the brain. Methodologies for probing each level, as well as the EEG markers we describe in this paper, are presented aligned with the biological level that they are hypothesized to capture. We also include bidirectional arrows; for example, local networks may both influence and inherit the activity at global network levels.

The following sections will consider common EEG markers (gamma power/frequency and beta power) of E or I or their interaction (E/I), before proposing 5 novel candidate markers (neuronal avalanches, long-range temporal correlations, entropy, microstates and the aperiodic 1/f signal), as well as identifying the hypothesized level of inference these metrics address. Figure 1 presents the levels of inference and the hypothesized level that each marker may assess.

## Conventional EEG markers of E/I in the living human brain

All neural oscillations, at any given frequency, rely on excitatory cellular activity. However, beta and gamma frequencies have been mechanistically linked within the literature to activity in excitatory cells and their interplay with interneuron activity [43].

### Gamma power and frequency (hypothesized level of inference: local population level)

Traditionally, high-frequency gamma oscillations recorded in the LFP, ECoG, and EEG have been used as putative noninvasive indicators of E/I [44–46]. Gamma oscillations (30–100 Hz) are rhythmic activity caused by synchronized fluctuations in the membrane potential of excitatory and inhibitory neurons [47–50]. After fast-acting excitation, there is a period of delayed feedback inhibition from PV interneurons (often GABAA interneurons are specifically implicated) that constrain cellular activity via a negative feedback loop [51–54]. The axon conductance of the synaptic delay leads to a phase shift between the spikes, which also determines the frequency of the gamma rhythm [54]. Increased inhibitory GABA (as measured by proton MRS) has been identified in line with increased gamma power in the superior temporal sulcus [55], supporting a causal role for interneurons in the generation of gamma activity. Also, the GABAergic drug propofol is known to increase gamma band power [56]. Modeling of oscillatory changes with propofol exposure indicates this effect is the result of increased inhibition within local circuits [57]. However, results from computational modeling suggest that peak gamma frequency has a stronger relationship with E/I function compared to gamma power [45]; moreover, visually induced peak gamma frequency is related to resting GABA levels—since the frequency of the gamma oscillation has been thought to reflect the time delay between fast excitation and the subsequently delayed inhibition [58–60].

Conversely, multiple studies have reported no association between GABA levels, gamma band power [58, 61], and gamma frequency [56, 61]. According to E/I models, pyramidal cell activity are a substantial generator of gamma oscillations; yet gamma rhythms are also dominant in neural areas such as the basal ganglia that lack local E/I connections [62, 63]. Yet, the latter could be seen as an expression of cortical dynamics. Thus, the mechanistic underpinnings of gamma oscillations are still unknown, and this makes interpretation of the mixed findings in psychiatric research difficult (e.g., refs. [61, 64–66].

### Beta power (hypothesized level of inference: local population level)

Beta oscillations (at ~13–28 Hz) are thought to be paced by networks of inhibitory interneurons and gated by GABAA action [43]. In this review, we recognize that there is a continuous frequency range for cellular firing, and frequency band cutoffs can be somewhat arbitrary. Nevertheless, we have taken beta oscillations as those occurring at ~13–28 Hz (as 28–30 Hz is considered low gamma). A computational model suggests that beta power increases due to a larger inhibitory drive onto inhibitory interneurons [67]. Although the theoretical link here is specific to inhibition, inhibition is a key ingredient in E/I flux. More specifically, somatostatin cell spiking is critical for generating a visually induced beta oscillation [68–70]. Beta frequency has therefore been likened to a state of central nervous system activation [71].

Further evidence linking beta oscillations to GABAA receptors comes from pharmacological studies targeting the GABAA system. Medications that enhance the GABAA system, such as benzodiazepines and compounds targeting alpha1 or the alpha2/alpha3 GABA subunits, are known to trigger an increase in EEG beta power in rodents and humans [67, 71–75]. Furthermore, evidence has suggested that the genetic marker of beta oscillations is located on chromosome 4 where a cluster of GABAA receptors are present [43]. Thus we conclude that beta power may reflect GABAA inhibitory neurons—and beta oscillations reflect inhibitory activity in local populations of neurons where GABAA neurons are prominent. Indeed, beta oscillations have been further differentiated into two types [43]. The first type of beta is thought to be generated by the phasic firing of excitatory pyramidal cells and inhibitory interneurons at beta frequencies. The second type of beta has been linked to synchronized pyramidal cells firing at beta frequencies with interneuron cells firing at gamma frequencies. The latter type of beta oscillations has been described as a subharmonic of gamma oscillations (e.g., refs. [43, 76].

However, although generated by local circuits, given extensive local and widespread connections throughout the brain, beta (and gamma) oscillations presumably both inherit and influence global E/I activity in the brain. Having outlined two conventional EEG markers that have been used to investigate E/I activity in EEG, the following sections will outline four novel EEG markers.

Whilst we have presented an overview of the literature linking gamma and beta power/frequency to E/ I activity and associated pharmacological studies, indeed, all oscillatory activity, regardless of the frequency band measured using EEG, is due to reciprocal interactions of excitatory and inhibitory neurons within reciprocal feedback loops. However, gamma and beta power have been more specifically investigated within the context of preclinical work and pharmacological studies examining how they are modulated. The specific cell networks have hence been described more thoroughly. Moreover, the modulation of gamma and beta power/frequency, as the result of a pharmacological challenge, may be the result of a frequency shift and an accompanying up or downregulation of oscillations in a separate frequency band. We encourage future research to focus more specifically on the network of up/downregulation across the frequency spectrum in response to pharmacological manipulation.

## Novel EEG markers of E/I in the living human brain self-organized criticality

One way to index E/I at the global brain level is to characterize specific brain-wide states that are associated with the relative or summed levels of excitation or inhibition. Excitatory and inhibitory dynamics have been implicated in maintaining the brain in a state of “self-organized criticality” [77]: this is a neural state dynamic centered at the boundary between two distinct regimes—(1) subcritical dynamics which are asynchronous, more dominated by short connections, quiescence, and noise, and (2) supercritical dynamics which are highly synchronized events characterized by run away excitation [78]. Self-organized criticality is associated with several important computational properties, such as long-range communication, and rich spontaneous dynamic brain states [79].

E and I are responsible for maintaining rich dynamical neural states that support the network’s intrinsic capacity (e.g., refs. [80, 81]. The evidence so far suggests that recurrent networks that are shaped by E/I plasticity mechanisms (i.e., the E and I firing patterns that govern the extent to which specific combinations of neurons fire together) tend to develop self-organized criticality [82, 83]. Further computational studies suggest that self-regulatory mechanisms modulating the activity of parvalbumin and somatostatin inhibitory interneurons should operate to keep the recurrent excitatory networks near the critical point of edge-of-chaos phase transition to dampen the effects of stimulus variability and ‘noise’ in the network dynamics [84, 85]. Moreover, critical states related to alpha oscillatory activity have also been suggested to optimize information transmission across cortical areas [86]. These considerations emphasize the importance of inferring E/I during experiments engaging the neocortex to be able to relate them to cognitive processes that might be transient and unfold quickly in time.

Pharmacological studies suggest that flux in E/I may dictate variations from criticality, i.e., chaotic flipping between three regimes—weak synchrony, criticality, and intense synchrony. Studies have used GABAA-receptor antagonist picrotoxin to reduce inhibitory synaptic transmission in leech ganglia [87] and rat cortices [88, 89] to show that inducing excitability leads to supercritical regimes in the form of strongly synchronized bursts [89] and exponential burst size distributions [87–89]. In addition, NMDA receptor antagonists APV [87] and AP5 [89] have been used to reduce excitatory synaptic transmission [88]; applied simultaneously with AMPA antagonist DNQX in [90] and lead to subcritical regimes in the form of weakly synchronized bursts [89] and bimodal burst size distributions [87, 89]. These results have also been replicated using computational modeling [89].

In addition, to stress the relevance of E/I balance to cortical dynamics, it is important to consider the fact that this state is actively maintained by cortical networks through homeostatic mechanisms [11]. Indeed, it has been demonstrated that pyramidal neurons scale the strength of their incoming glutamatergic synapses after perturbations in excitatory drive, to maintain stable firing rates [91–94]. Not only that, but later studies have shown that the homeostatic maintenance of E/I balance further involves the regulation of intrinsic excitability [95] and incoming inhibitory synapses [31, 96–98], likely originating from PV interneurons [94]. In addition, recent results show that sensory deprivation, which effectively decreases the levels of excitatory drive to the visual cortex and disrupt its E/I balance, also causes a significant departure of dynamics from the critical regime [99]. Moreover, criticality was returned in a timescale of days, likely through the same homeostatic mechanisms that ensure stable firing rates. Therefore, the aforementioned studies are important not only because they show that cortical networks actively regulate their E/I balance to compensate for perturbations in an external drive, but, more importantly, because they pose criticality as a homeostatic setpoint of cortical dynamics. Therefore, current knowledge of E/I homeostasis lends support to the hypothesis that criticality is a relevant biomarker of E/I balance.

In the following sections, we describe four markers that categorize critically, or phase states, within EEG, and how these metrics might index the E/I system. We also provide references for calculating these metrics and inferring criticality from the EEG spectrum. We then go on to describe one final marker, which has been examined outside of the context of criticality, and its potential relationship to E/I.

### Neuronal avalanches and the Kappa coefficient (hypothesized level of inference: global network level)

Criticality exists at the border between asynchronous and intensely synchronous systems. In the brain, self-organized criticality might be inferred by measuring the spatial scaling and temporal fluctuations that evolve due to long‐range temporal correlations. An important hallmark of self-organized critical systems is summary measures of dynamics displaying power laws [77, 90]. Indeed, neuronal avalanches are defined by having fractal-like scale-free properties characterized by a power-law structure. Computational models have supported the relationship between E/I flux and scale-free network dynamics [100]. With EEG therefore, neuronal avalanches have been used as an indirect index of criticality (or fluctuations from) [88, 89, 101].

Neuronal avalanches observed in vitro [88], in vivo [102], or from scalp EEG and MEG [103], are cascade-like events that increase and advance over a system—akin to a successive wave of action potential firing from neuron to neuron. Neuronal avalanches demonstrate a characteristic distribution of burst sizes that follows a power law with an exponent close to −1.5 [88, 89, 101, 103, 104]. They can be measured in EEG by identifying very high amplitude periods of activity that are clustered within a particular brain region and time window [88, 103, 105]. This involves some measure of the size of the neural signal on the *x*-axis, such as the physical extent of patterns of activity in, for example; LFP, voltage-imaging, BOLD fMRI, or EEG/MEG activity. Computational models [106, 107] indicate that optimal inhibition promotes critical dynamics and that a lack of inhibition modulates the time length and size of avalanches [108]. This same modulation has been demonstrated with pharmacological increases in inhibition using propofol [109].

Another way of inferring whether the brain sits at criticality at a specified timescale is to analyze the extent to which avalanche dynamics follow a 1/f power law within a set recording period. The kappa coefficient [80] is a non-parametric measure of the goodness of fit of the data of interest to a given power law. Kappa (k) is calculated by comparing the cumulative density function of the measured data with a theoretical reference cumulative density function (−1.5) to assess the strength of fit with a power law. In the context of LFPs, pharmacological studies with glutamatergic or GABAergic agonists in vitro have shown that k ≈ 1 at criticality, and is >1 or <1 in super- or subcritical dynamics, respectively [87, 89, 101]. Hence, neuronal avalanches could provide a useful characterization of self-organized criticality, and, therefore, E/ I balance. Using the kappa coefficient could further help to elucidate evidence of E/I imbalance in certain populations or under certain conditions.

### Long-Range Temporal Correlations (LRTC) and functional EI balance (hypothesized level of inference: global network level)

In addition to power-law distributions of avalanche sizes on a global scale, signatures of criticality emerge at the level of local network oscillations. More specifically, due to their scale-free nature [110–112], critical systems show self-affinity in their activity patterns. This means that the statistical properties (e.g., standard deviation) of the same signal observed within two windows of different sizes can be related through a scaling parameter *L*^*H*^, where *L* represents the ratio between the lengths of the windows and *H* is commonly known as the Hurst-coefficient [113–115]. While cumulative signals emerging from purely stochastic processes have Hurst-Coefficients close to 0.5, systems with LRTC, are characterized by 0.5 < *H* < 1 [115]. Thus, in such systems, where activity at a certain time point has a level of dependence on the previous activity (i.e., systems with memory), we observe larger fluctuations on longer-time scales than would be expected from a signal generated by a purely random process. This is relevant, because not only has it been shown that critical systems, being scale-free, exhibit LRTC [111, 112], but also that this is a property of neural network oscillations measured, for example, through EEG signals [100, 111]. In addition, modeling work has demonstrated that optimal E/I balance simultaneously leads to the emergence of avalanches with a −1.5 power-law distribution in spiking activity and also the emergence of LRTC in the amplitude of oscillations in the alpha/low-beta frequency range [116].

Given the evidence towards long-range temporal correlations as a biomarker of criticality, and thus E/I balance, it is relevant to consider methods to quantity them in oscillatory signals. In that regard, detrended fluctuation analysis (DFA), developed by Peng and colleagues in 1994 [117], has been used extensively to measure the scale-free nature of physiological signals. DFA uses a cumulative time series of fluctuations in the amplitude of oscillations in frequency ranges of interest to estimate Hurst-coefficients [115]. Therefore, a signal with a DFA exponent between 0.5 and 1 is considered to show LRTC. While this type of analysis has extensively shown the existence of LRTC in alpha- and beta-range oscillations in human electrophysiological data [100, 111, 118–120], the same behavior has not been observed in gamma oscillations, likely due to the reduced data quality of such oscillations in E/MEG signals [115]. In addition, this marker has been linked to neurological conditions related to altered E/I balance. In patients with major depressive disorder, it was observed that DFA exponents inversely correlated with symptom severity [121]. In addition, a breakdown of LRTC at alpha and beta frequency bands has been further observed in patients with Alzheimer’s disease [122] and schizophrenia [123], which have been previously associated with E/I dysregulation [4, 5, 124]. Conversely, in epileptic patients, elevated levels of LRTC are observed in areas close to the epileptic focus [125]. However, one of the caveats of DFA is that the information it provides is limited to how far a system is from criticality, regardless of whether it is sub- or supercritical. To solve that, a recent method was developed to infer the state of dynamics relative to the critical point through an adaptation of DFA [100]. Besides validating their metric of functional E/I balance using a computational model of a population of E and I neurons, the authors further applied it to infer differences in local E/I balance between autistic and non-autistic individuals.

In conclusion, the observation of LRTC in human electrophysiology further supports the hypothesis that criticality is a robust property of cortical activity and a relevant biomarker of E/I balance. While the use of DFA to infer critical dynamics has been validated, both in the typical [111, 118–120] and atypical brain [122, 123, 125, 126], only recently was it extended to inform on the exact nature of network E/I balance (i.e., excitation or inhibition dominated) [100]. Therefore, further studies should be conducted to evaluate the validity of such metrics to be used as biomarkers of local E/I balance and how they reflect changes in local network dynamics in pathologies that have been associated with E/I imbalances.

### Neural entropy (hypothesized level of inference: global network level)

In information theory, entropy (typically measured in computational units such as bits) quantifies the amount of information within a signal [127] as well as the consistency of the neural population activity. Entropy is large when there is a more variable pattern repertoire) and small when there is less variable pattern repertoire. It has been posited that information capacity is a functional property maximized in a balanced and critical cortex [79]. Likewise, others have hypothesized that the variability of burst area would also be maximal under the same E/I conditions that produce neuronal avalanches [89].

The same experiments cited above, which manipulated E/I balance in vivo using pharmacological manipulations [80, 89], observed peak entropy of spontaneous [80], evoked [80], and burst activity [89] in the no drug conditions when E/I are assumed to be balanced, neural avalanches are observed and k ≈ 1: that is, peak entropy is observed when the brain is at criticality. These in vitro findings are further corroborated by in vivo evidence from a study of two awake monkeys and six urethane-anaesthetized rats [80], and computational models [80, 89]. In addition, it has previously been reported that information capacity dynamically tracks the recovery of mice from anesthesia, peaking when an awake state is reached [128]. The same entropy-tracking effects of anesthesia have also been observed in human EEG where anesthesia was induced with sevoflurane and propofol [129]. It has been noted that whilst higher entropy is exhibited by networks with balanced E and I vs. imbalanced E and I, there are many ways in which networks could achieve balance depending on E and I synaptic strength and quantity. One study concluded from their computational model that, within balanced networks, stronger synapses lead to an intermediate entropy that is resilient to subtle alterations of the system that may be more favorable for mammalian cortices [130].

Taken together, this evidence suggests that peak entropy of spontaneous, evoked, or burst EEG activity is a consequence of balanced, critical systems and thus can be used to indicate fluctuations in criticality and E/I balance. However, it may be argued that this is somewhat expected—i.e., that entropy tracking can be used to observe the arousal peak following recovery from anesthesia, given that anesthesia often produces high amplitude, but low-frequency waves. By default, we would expect lower extropy values since these waves contain less information than a conscious brain. However, appreciating this explicitly within the scientific community may still be somewhat informative, and entropy may be a useful measure if it correlates more specifically with independent network activation. However, we appreciate that entropy may not be independent of power analysis and neural frequency shifts.

Despite their promise, the definition of neuronal avalanches and entropy measures in multichannel EEG/MEG is still evolving—the high dimensionality of the data and the dependencies of scale-free dynamic computations of these dimensions make it challenging to comprehensively understand the mechanisms underlying both criticality and entropy. Further research is needed to understand these measures and their relationship to excitatory and inhibitory networks.

### Microstates (hypothesized level of inference: global network level)

Relatedly, changes in phase state might also index E/I flux. Such changes might be captured by EEG microstate activity: short durations (~100 ms) of relative stability in EEG scalp topography [131]. In microstate analysis, ‘brain states’ refer to the topographic stability of electric potentials over an electrode array. Neuronal oscillations demonstrate intervals of phase-locking, before transitioning to new phase-locked states [132]. Microstates may thus reflect neuronal coordination and the dynamic range of neural activity [133]. Indeed, EEG microstate class has been found to covary with the spatial distribution of thalamic activity in an EEG-fMRI study [134].

Evidence to support the relationship between EEG microstates and E/I balance comes from a study whereby EEG was recorded from eight healthy adults who received either 30 ug of Lorazepam (a GABA agonist) or a placebo. Results found that the amount and intensity of structurally synchronized microstates increased with Lorazepam [135]. Further to this, recent research decreased cortical excitability using low-frequency (fewer bursts per second i.e., ≤1 Hz) repetitive transcranial magnetic stimulation (rTMS). The results showed an increased mean duration, immediately after rTMS (for microstates A, B, and C) and 1 h after rTMS (for microstates C and D; [136]: that is, the average time of consecutive time frames differed for these microstates. The authors interpreted these findings as evidence for increased stability of microstates post-rTMS (i.e., fewer transitions). Finally, researchers have utilized simultaneous, multi-modal recordings of PET, MRS, and EEG in 29 healthy subjects. Data were collected at the resting state, and results demonstrated a small significant positive correlation between source-localized microstate measures and GABAA-receptor availability [137].

### Aperiodic 1/f signal (hypothesized level of inference: global network level)

A final promising candidate marker of the relative balance of E/I is the aperiodic signal of the power spectral density slope (PSD)—often referred to in the literature as the ‘1/f component’. Note, here, 1/f denotes the behavior of EEG data in the frequency domain such that as frequency increases, power decreases. Gao et al. leveraged a simple computational model of the EEG signal composed of non-oscillatory excitatory (AMPA) and inhibitory (GABA) currents. These currents are significant contributors to the EEG [13], and when they are integrated, they naturally give rise to the aperiodic 1/f slope. To test this they varied E/I ratio from 1:6 to 1:2 [138] and measured the effect on the simulated aperiodic 1/f slopes. The E/I function was defined as mean excitatory and inhibitory conductance over simulation time. The authors observed that reduced inhibition flattened the slopes.

In the same paper, shank recordings (voltage membrane recordings of postsynaptic excitatory cells) from rodents were analyzed, obtained from Collaborative Research in Computational Neuroscience (CRCNS) data portal [139], sampling the LFP at evenly spaced electrodes of the pyramidal cell layer in CA1. Aperiodic 1/f slopes were then estimated by fitting a linear regression slope between 30 and 50 Hz across CA1 depth; the E/I function of the cell layers in CA1 was estimated based on previously published synapse density values [140]. The authors demonstrated that the aperiodic 1/f slope varied with estimated AMPA to GABA synapse ratios in CA1 layers. The authors also reanalyzed open-source electrocorticogram data from macaques undergoing propofol sedation (a period of increased inhibition) and demonstrated that the aperiodic 1/f slope became more negative during sedation [90].

This aperiodic 1/f signal can be computed by assuming that the power spectrum has the form within a certain frequency range, usually above a characteristic “knee” frequency, below which the power-law relationship is different (if present at all) [141]. When this relationship is plotted in log-log space, the exponent (-n) of the slope refers to the steepness of that line. Whilst the relationship between power and frequency is often referred to as a 1/f power law, n can have various values, typically ranging between 0 < n < 2. However, in the study of E/I this method requires the 1/f fit to be performed in the aperiodic component of the signal—after removing oscillatory parts corresponding, for example, to alpha and theta peaks [142, 143]. We suggest that going forward, researchers should perform this step to ensure it is the aperiodic component specifically that is linked to E/I, instead of the relationship being driven by up or down regulations in EEG power within specific frequency bands.

Overall, self-critically has been theorized to underlie the emergence of power laws across many physical systems [144]. Indeed, 1/f power spectra and long-range temporal correlations are known properties of critical systems [110, 115], and have been simultaneously observed in electrophysiological data [111], suggesting that they could both reflect the criticality of human neural dynamics. However, crucially, the presence of a power law alone does not imply criticality. Tests of criticality in the cortex require a means of experimentally manipulating neuronal interactions and the ability to evaluate when the cortex is at criticality [79]. Furthermore, multiple alternative explanations could underlie power-law generation in EEG power spectrums. One group demonstrated that by modeling electrophysiological data as a collection of damped oscillations that are randomly perturbed and fade away with different relaxation rates, they were able to explain its 1/f nature [145]. Alternatively, others have argued that the filtering properties of extracellular media can explain 1/f power-law generation [146].

Nevertheless, the evidence here suggests that the aperiodic signal in human EEG may be a useful marker of E/I balance within the brain. It assumes that background activity makes up the “backbone” of the LFP (or ECoG/EEG) and that this activity is asynchronous, derived from summed Poisson population firing—it thus would reflect more global dynamics [147]. For guidance on how to compute the aperiodic 1/f component see ref. [143]. The model of the EEG power spectrum independently estimates the (1) periodic signals, of phase-locked oscillatory activity in specific frequency ranges i.e., beta, gamma etc., and (2) aperiodic component, that is; a signal which does not repeat itself after a specific time period [143].

## Discussion

### Limitations, future work, and marker development

In this article, we have highlighted a variety of EEG metrics that may tap into the E/I system. For guidance on how to extract/ analyze, each of these EEG markers, see Table 1. These markers may offer translational potential since they can be applied in tasks that do not require verbal ability, or extensive cognitive processing - allowing us to examine brain function between species and all individuals within a species. However, measures of beta power are particularly promising given the wealth of studies across species (rodents, non-human primates, humans) with benzodiazepines and newer compounds that are selective for the GABAA receptors containing α1–3 subunits. Nonetheless, newer metrics also offer promise (e.g., 1/f; entropy; microstates). Yet, each of the markers presented here is likely intercorrelated with one another, and potentially measures overlapping aspects of the same underlying signal—this is a challenge inherent in all EEG analysis—and perhaps in human sciences more generally. When aiming to map a specific marker or variable to a specific mechanism or cognitive process in isolation, one potentially ignores the variable’s relationship to a range of other related variables and factors. Furthermore, a second challenge is mapping each marker to a specific level of inference (i.e., the micro or meso-level). All brain activity across micro-, meso-, and macro-levels of the organization is the result of excitatory or inhibitory firing on a continuous scale. Local networks will indeed influence global brain dynamics, and local networks may inherit properties of global brain dynamics. However, specific markers and areas of interest can be used to zoom in and out to capture the resulting activity at the local or global level. This, of course, does not mean that the activity is modular—just that this activity can be captured and investigated at various levels. This approach will also lead to a better understanding of how local activity and global activity interact.

We note that whilst there have been correlations reported between state (critical or phase) change and some aspects of E/ I flux, the exact mechanism for this relationship has not been well documented. Analysis of entropy, kappa, and microstates as proxy markers of E/I comes with caveats. The research is in its infancy, and although correlations have been reported, thorough validation has not taken place. We hypothesize that multiple mechanisms could underlie this correlation. Changes in brain state—for example, from synchronous to asynchronous, or substantial changes between oscillatory phases, may result from GABA-mediated thalamocortical circuit inhibitory bursts [148, 149]. Feedforward thalamic inhibition disrupts synchronous pyramidal cell firing and may trigger a change in the frequency of oscillations—thus also altering E and I activity in the cortex. Imaging studies in humans have shown that propofol, a GABAA-receptor agonist, disrupts thalamocortical connectivity [150]. This activity may then alter local E and I interactions in the cortex. One example of this is the generation of the alpha rhythm in the cortex during anesthesia, which is accompanied by changes in the interaction between the thalamus and cortex. This may be indexed globally by looking at whole-brain metrics of state change e.g., criticality analysis, microstate analysis, and by documenting entropy change – but these markers need more comprehensive interrogation in combination with drug challenge studies that alter thalamocortical communication.

Indeed, many of these markers have already been investigated within clinical populations in an attempt to investigate E/I (see e.g., refs. [64, 151, 152]. However, a lack of understanding of the precise mechanism underlying these markers has led to contradictory findings (see ref. [10]), and as yet, there is no EEG marker of E/I that has been formally validated nor accepted according to FDA and/or EMA biomarker specification. Further work in this area is needed, as well as clearer descriptions of the mechanisms thought to underlie each marker.

Therefore, before further inference is made using these markers across psychiatric and neurodevelopmental disorders, further work would be needed to develop and examine them as biomarkers of E/I—this would involve further interrogation of the mechanistic link between each marker and E/I. It would also be essential to assess the markers' accuracy, reproducibility, reliability, and utility. Confidence in the accuracy of a marker involves producing a high level of converging evidence, ideally from independent laboratories (i.e., a replication sample), all demonstrating that a proxy marker indexes E/I balance. For example, an association between these markers and the E/I system can be made by combining a marker with pharmacological studies that modulate the level of inhibition and excitation [153, 154]. Computational work in this area is largely lacking but would be particularly important for identifying mechanisms and potentially bridging together preclinical and clinical work. There is also a potential for human induced pluripotent stem cells (iPSC) cell work to bridge this gap—iPSC models provide reductionist 3D models of brain anatomy. Initial evidence has highlighted the role of E/I in initiating and maintaining iPSC oscillations [155]. Examining the EEG metric readouts on iPSC oscillatory activity, where the GABAergic and glutamatergic networks are more specifically defined, may allow us to interrogate more causative associations between E/I and a particular metric.

The identified markers should also be sensitive and specific—i.e., sensitive to changes in E/I above and beyond any other general brain metrics—this is a hard condition to meet given some of these markers may correlate i.e., gamma and beta power. However, machine learning (e.g., using feature selection or decision trees) can help us identify which EEG markers may help us predict another E/I measure, for example, GABA from MRS. Test-retest metrics are necessary to ensure the validity and reliability of each marker. Finally, future work should also aim to distinguish between ‘loosely’ (i.e., on average) balanced systems, and systems that demonstrate a rigorously controlled balance between E and I [156, 157]. Markers that then fulfill these criteria could be used for patient selection in interventional trials, and also for tracking pharmacodynamic effects in pharmacologic treatment trials.

An alternative possibility is that an observed E/I balance is a consequence of atypical structure within any level of the neuronal system. Any variation in neural structure or synaptic function will likely have implications for neural function (i.e., E/I) since neural cells are largely either inhibitory or excitatory. This latter possibility is consistent with recent evidence that alterations in E/I function can be compensatory [158]. Indeed, it has been suggested that highly disruptive events such as stroke lesions, which cause an acute loss of excitation to cortical neurons across the brain, may trigger E/I homeostatic mechanisms to compensate for this disruption and recover cortical function. Interestingly, this physiological response might be related to late-onset symptoms of stroke, such as epilepsy and chronic pain, thought to be influenced by alterations in E/I balance [159]. Regardless of whether E/I is a cause or consequence of a neuropsychiatric condition, capturing variance in the E/I disruption may provide key information about the extent to which a neuronal system is affected, and in which individuals. For example, a subset of individuals may indeed show alterations in E/I activity which is known to be altered by a specific drug, - this may indeed be a promising treatment target for that individual should the individual need or require treatment for a particular symptom i.e., subclinical epileptic features. The markers may also have utility for determining which individuals have subclinical epilepsy or may go on to receive a diagnosis of epilepsy. All these hypothetical scenarios will need rigorous testing.

## Conclusion

There is a need for noninvasive and simplified metrics that can measure perturbations in neural activity, and that can signal brain health in a variety of neurodevelopmental and psychiatric conditions. Importantly, we currently do not have any validated noninvasive methods for examining E/I imbalance in humans; however, a variety of potential candidate proxy markers derived from EEG offer promise. Many of these markers are interconnected. For example, beta/gamma power will be influenced by the slope of the PSD spectrum (i.e., the extent to which it varies from 1/f) [143]. Because many of the markers are correlated or conflated, it is important to also consider each E/I marker in terms of its biophysical plausibility, to ensure that the intended feature is being properly measured [160]. However, examining each marker in parallel will provide a richer description of brain dynamics and excitatory versus inhibitory properties of the network. Validation of each marker will open the gate toward capturing E/I mechanisms in a variety of neurodevelopmental and psychiatric conditions. The next steps would be to explore how individual differences in the E/I function might explain trait/ phenotypic variation within and between conditions, or how E/I brain dynamics vary across development.
